# Experimental and numerical study of stick–slip phenomenon in granular materials

**DOI:** 10.1038/s41598-025-13859-7

**Published:** 2025-08-01

**Authors:** Justyna Sławińska-Budzich, Barbara Świtała

**Affiliations:** Department of Geomechanics, Institute of Hydro-Engineering, Polish Academy of Sciences, Kościerska 7, 80-328 Gdańsk, Poland

**Keywords:** Stick–slip, Discrete element method, Force chains, Plane strain conditions, Civil engineering, Mechanical engineering

## Abstract

The stick–slip is a characteristic phenomenon of various mechanical systems, occurring when two bodies slide relative to each other. Friction plays a dominant role in this phenomenon, transitioning from static to dynamic when sudden movements take place. Studying this behaviour is particularly important for understanding stress transfer in granular materials. The study examines the development of the stick–slip phenomenon under plane strain conditions, which are commonly encountered in large-scale geotechnical structures. Experiments performed on glass bead samples investigate the effects of varying confining pressures, shear rates, initial void ratios, and degrees of bead wear on the development and characteristics of the stick–slip phenomenon. Additionally, a plane strain triaxial test is simulated using the discrete element method (DEM). Although the stick–slip events are not fully reproduced, some micro-slips are identified in the results. The physical mechanism underlying their development appears to correspond to the standard stick–slip behaviour reported in the experiments. Since micro-mechanical behaviour is a key to understanding the stick–slip phenomenon, incorporating DEM analyses opens a new route for determining micro-parameters and linking them to macro behaviour.

## Introduction

The stick–slip phenomenon in granular materials refers to the movement of two contacting surfaces (grains), characterised by two distinct phases: stick, which is a static or nearly static contact, and slip, which is a sudden movement, sliding between the surfaces.

This phenomenon and the conditions of its occurrence depend on several factors, including the friction between the surfaces, the size and shape of the particles, the void ratio of the granular sample, the magnitude and nature of the forces acting on the assembly, and the velocity at which the force is applied^1–3^. In granular assemblies, stick–slip behaviour is often attributed to the reorganisation of force chains and the collapse of loops that support them^4–6^.

The research related to this aspect is important in geosciences and other engineering disciplines, including civil engineering, material science, tribology, and the pharmaceutical industry. It may affect processes in which granular materials are present such as landslides (slope stability)^7,8^, avalanches^9^, silo discharge^10,11^, pharmaceuticals and powder materials management^4,12^ and foundation design. Furthermore, stick–slip behaviour during shearing in tectonic fault zones is considered a potential source of earthquakes^13–18^.

In geotechnical engineering, the stick–slip phenomenon has been studied through laboratory tests, field observations, and numerical modelling. The findings from these investigations can be applied to real-world scenarios to better understand the conditions that lead to stick–slip occurrence. The phenomenon has been experimentally investigated primarily through classical triaxial tests^19–25^. Cui et al.^22^ examined the influence of various factors on its development, including shear rate, a wide range of confining pressures (50–1000 kPa), and the degree of wear. Doanh et al.^23^ tested saturated samples under drained conditions, considering variable shear rates at different confining pressures. Similar research was conducted by Ozbay and Calabar^24^. Xu et al.^25^ performed experiments under both drained and undrained conditions. Daniels et al.^26^ conducted experiments demonstrating that the stick–slip phenomenon is correlated with voltage signals. More recently, Liu et al.^27^ studied stick–slip development using a direct shear apparatus, while Gou et al.^28^ investigated the phenomenon using a ring shear apparatus, including an analysis of acoustic emissions.

Zhuang et al.^29,30^ examined the behaviour of glass bead samples in plane strain conditions. They tested two types of sand and two types of glass beads. The digital image correlation method was employed to track the evolution of strain within the samples. Their experimental results showed that particle shape, relative density, confining pressure, and the selected stress path significantly influence shear behaviour. Many analyses have been conducted, but no attempt was made to quantify stick–slip. Nonetheless, they qualitatively determined that this phenomenon occurs and becomes more pronounced at higher pressures. Therefore, in this paper, we focus on the quantitative description of stick–slip events in glass bead samples under plane strain conditions, considering the influence of various test and material parameters on their development, including the magnitude and frequency of event occurrence. Specifically, we examine a wide range of confining pressures (200–800 kPa), different shear rates, initial void ratios, and degrees of wear. Our tests have confirmed that plane strain conditions favour the development of the stick–slip phenomenon. This is particularly significant given that such conditions are commonly encountered in large-scale geotechnical structures such as embankments, dams, and retaining walls.

Based on experimental results, a unique database has been established, enabling a better understanding of the nature of the stick–slip phenomenon and the conditions favourable for its development. These fundamental observations provide a broader perspective on the problem and offer potential for scaling up to more complex conditions. Furthermore, such tests provide a rich database for the validation and calibration of numerical models.

Numerical analyses of the stick–slip phenomenon are quite limited. Discrete Element Method (DEM) simulations have been conducted by Ferdowsi et al.^31^, who investigated micro-slips as precursors to larger slip events characteristic of fault zones. To study this behaviour, a direct shear test was modelled. Bai and Konietzky^32^ conducted a DEM analysis of a plane joint, enabling quantification of the energy balance during stick–slip. The DEM simulations of triaxial tests, in which the stick–slip occurrence was reported, have recently been performed by Huang et al.^33^. They modelled the behaviour of samples at relatively high pressures of 1000 kPa and 2000 kPa. Hazzar et al.^34^ simulated drained triaxial tests on glass-bead samples and obtained stick–slip events, but have not commented on them. Furthermore, granular assemblies were subjected to triaxial compression in DEM simulations in the paper by Jiang et al.^35^. The analysis revealed that the structure and evolution of force chains depend on the degree of saturation.

Given that existing studies do not comprehensively integrate both experimental and DEM approaches in investigating the stick–slip phenomenon, the present paper attempts to model stick–slip events numerically. Since micro-mechanical behaviour is considered fundamental to understanding this phenomenon, incorporating DEM analyses offers a promising route for identifying micro-parameters and linking them to macro-scale behaviour. Although the applied model did not fully reproduce the experimental stick–slip events in terms of magnitude and frequency, certain micro-slips were observed. The physical mechanisms underlying their development appear to correspond to the typical stick–slip behaviour seen in experiments. The conclusions drawn from the numerical analyses are noteworthy, for instance, the observed increase in the number of stick–slip episodes with higher friction coefficients or larger bead radii. Furthermore, the DEM model enables a series of sensitivity analyses, which help to assess the influence of various model parameters and visualise the force transmission paths between grains in the considered cases. Nonetheless, it is important to acknowledge the model’s limitations and assumptions, particularly those affecting the quantitative reproduction of stick–slip events in triaxial tests, which are discussed in detail in the following sections.

This paper is organised as follows. In “Methodology”, the methodology of the proposed research is described. Section “True triaxial tests” presents and analyses the experimental campaign. First, the test procedure is outlined, followed by the presentation of results from tests on glass bead samples. Various cases are considered to assess the influence of different material and test conditions on the resulting strength envelope, as well as the frequency and amplitude of developed stick–slip events. To this end, samples with different degrees of glass bead wear, initial void ratios, confining stresses, and shear rates were tested.

Numerical simulations are described in “Numerical simulations”, where a comparison between numerical and experimental results is also presented, highlighting the model’s limitations. This comparison is followed by a sensitivity analysis that investigates the influence of various shear rates, friction coefficients of glass beads, and grain sizes on the results. Furthermore, micro-mechanical analyses of the numerical sample are performed, enabling a detailed investigation of the sample’s microstructure. Finally, “Conclusions” concludes the paper.

## Methodology

The true triaxial apparatus is used to determine the shearing characteristics of glass beads in plane strain conditions. The discrete element method (DEM) is employed in numerical analyses.

Experiments performed in the laboratory in the true triaxial apparatus enable the identification of shearing characteristics of the considered material. This kind of test gives the possibility to examine the behaviour of the material in the complete stress space, whereas in a classical apparatus, only a partial stress space is available and the condition $\sigma _{2}$ = $\sigma _{3}$ is enforced. In the true triaxial tests, samples are cuboid with dimensions 150 $\times$ 75 $\times$ 75 mm. Plane strain conditions assume that deformation of the sample is prevented in one of the directions ($x_{2}$).

The Discrete Element Method (DEM) is further utilised to model the plane strain triaxial test numerically, allowing for the extraction of additional information and the study of force chain networks and their development in three dimensions. Such observations would be challenging to achieve experimentally or would require advanced, time-consuming experimental techniques. The DEM was developed by Cundall and Strack^36^ and has since gained popularity among researchers across various disciplines. In a granular assembly, each grain is treated individually and follows Newton’s Second Law of Motion, enabling the modelling of both translations and rotations of grains within the assembly. Given that soil consists of grains of different sizes and shapes, DEM has attracted significant attention in geotechnical research. Along with some basic laboratory tests,^34,37^, more complex problems have also been modelled^38,39^.

## True triaxial tests

### Test procedure

The samples were prepared in a rubber membrane stretched inside a specially designed mould. The beads forming a loose sample were piled up using the air pluviation method. The funnel with a certain mass of beads was inserted into the mould and then slowly lifted, remaining in contact with the material already placed into the membrane. The compacted samples were formed by pouring the weighted beads from a given height.

The characteristic parameters of the tested glass beads are listed in Table 1, where: $d_{50}$ is the particle diameter, which together with smaller ones contains 50% of the material mass, $C_{u}$ and $C_{c}$ are uniformity and curvature coefficients, respectively, $\rho _{s}$ is the grain density, $\rho _{min}$ and $\rho _{max}$ are minimum and maximum bulk density and $e_{min}$ and $e_{max}$ stand for the minimum and maximum void ratio, respectively.

**Table 1:** Physical properties of glass beads used in the tests.

Property	Value	Unit
Grain diameter	0.75–1.25	mm
$d_{50}$	1.1	mm
$C_{u}$	1.24	–
$C_{c}$	1.01	–
$\rho _{s}$	2.65	$\hbox {g/cm}^{3}$
$\rho _{min}$	1.541	$\hbox {g/cm}^3$
$\rho _{max}$	1.684	$\hbox {g/cm}^3$
$e_{min}$	0.574	–
$e_{max}$	0.720	–

In the first stage, the isotropic compression was applied until a given value of a confining pressure was reached. Then, the material was sheared in plane strain conditions, assuming various combinations of test conditions, listed in Table 2. All results are presented in the soil mechanics sign convention, where the plus sign indicates compression.

**Table 2:** Initial and characteristic test conditions.

Experiment	Degree of wear	$e_{0}$	$\sigma _{3}$ (kPa)	*v* (mm/h)
s11	Virgin	0.673	200	8.55
s13	Virgin	0.663	400	7.61
s16	Virgin	0.660	800	7.55
s17	Virgin	0.663	600	7.56
001/24	Virgin	0.650	800	122.87
002/22	Virgin	0.687	800	16.39
003/22	Virgin	0.603	800	15.70
004/22	Virgin	0.663	800	15.00
007/21	Virgin	0.689	700	14.94
008/21	Second use	0.693	700	15.38
009/21	Third use	0.700	700	15.63
010/21	Fourth use	0.694	700	16.13
011/21	Virgin	0.688	800	15.50
012/21	Second use	0.668	800	15.67
013/21	Third use	0.662	800	15.89
014/21	Fourth use	0.670	800	15.81

**Fig. 1:**
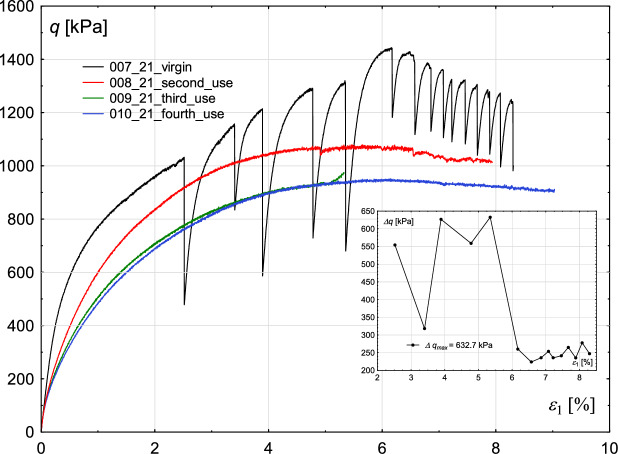
Influence of the degree of wear of glass beads used in the experiments. Deviatoric stress *q* (main figure) and deviatoric stress amplitude $\Delta q$ (inner figure—for cases in which stick–slip events develop) with increasing axial strain $\epsilon _{1}$ for the constant value of confining pressure $\sigma _{3}=700$ kPa.

The following experimental conditions were varied in subsequent tests (Fig. 1):

- degree of wear of glass beads (number of shearing events on the beads),
- sample’s initial void ratio,
- confining stress level,
- shear rate.

### Experimental results

First, the influence of the number of test repetitions on the strength of the sample and the development of stick–slip events is investigated. Strength is defined as the value corresponding to the envelope, excluding any deviator stress drops. This terminology is consistently used throughout the article.

### Degree of wear of glass beads

First of all, the influence of the degree of wear of glass beads on the results was tested, considering two values of confining pressures: 700 kPa and 800 kPa. The entire test procedure was repeated four times on the same set of glass beads. The stress-strain characteristics are presented in Figs. 1 and 3. The small, inner figure shows the changes in the amplitude of consecutive deviator drops. The initial void ratio and shear rate were kept similar for each test.

The results revealed a clear dependence of the occurrence of stick–slip events on whether the beads were used for the first time or had been previously used in earlier tests. In the case of a confining pressure of 700 kPa, stick–slip events developed only in the test where completely new glass beads were used. The overall strength of this sample was also significantly higher than that observed in tests with previously used beads.

**Fig. 2:**
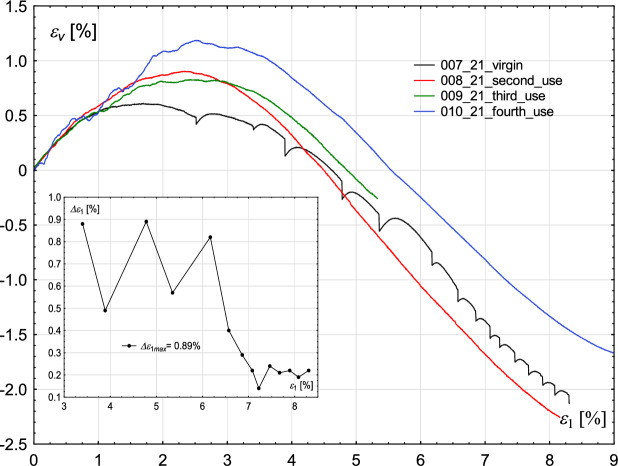
Influence of the degree of wear of the glass beads used in the experiments. Volumetric strain $\epsilon _{v}$ (main figure) and oscillation amplitude $\Delta \epsilon _{1}$ (inner figure—for cases in which stick–slip events develop) with increasing axial strain $\epsilon _{1}$ for the constant value of confining pressure $\sigma _{3}=700$ kPa.

**Fig. 3:**
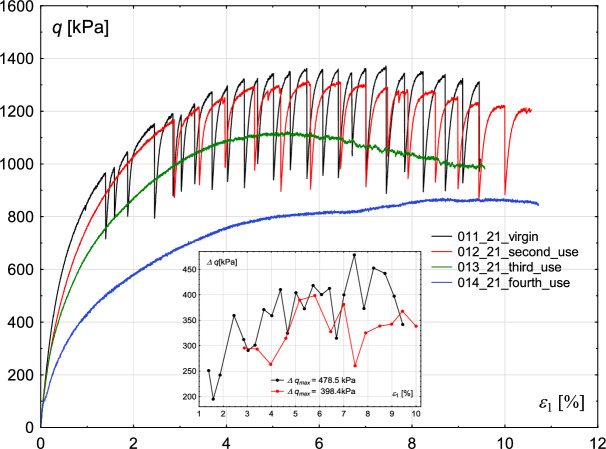
Influence of the degree of wear of the glass beads used in the experiment. Deviatoric stress *q* (main figure) and deviatoric stress amplitude $\Delta q$ (inner figure—for cases in which stick–slip events develop) with increasing axial strain $\epsilon _{1}$ for the constant value of confining pressure $\sigma _{3}=800$ kPa.

**Fig. 4:**
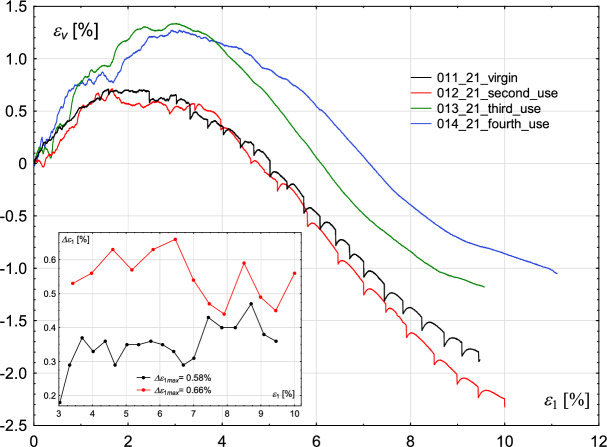
Influence of the degree of wear of the glass beads used in the experiments. Volumetric strain $\epsilon _{v}$ (main figure) and oscillation amplitude $\Delta \epsilon _{1}$ (inner figure—for cases in which stick–slip events develop) with increasing axial strain $\epsilon _{1}$ for the constant value of confining pressure $\sigma _{3}=800$ kPa.

**Fig. 5:**
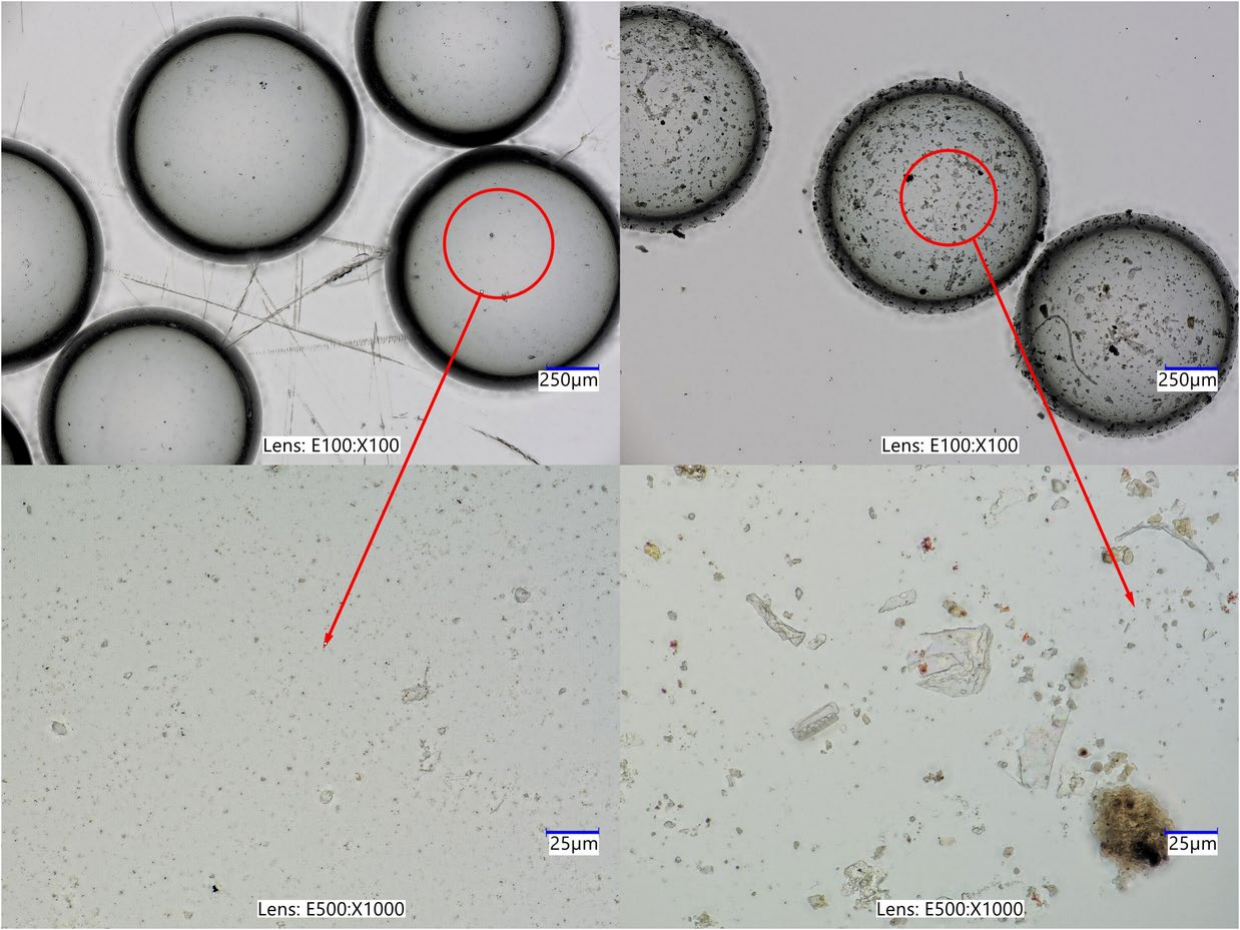
Microscopic observations of virgin (left) and reused (right) glass beads. Images in the top row show $100 \times$ magnification, while those in the bottom row display $1000\times$.

The amplitude of the rapid drop of the deviatoric stress in two alternating stick–slip events increases and decreases until the maximum strength of the material is reached. After achieving a strain level corresponding to the maximum strength, the amplitude of rapid drop decreases drastically (Figs. 2, 3, 4, 5).

Similar observations can be made for the case of a confining pressure of 800 kPa. However, in this series of tests, stick–slip events developed in the sample built of new beads, and in the test with beads used once. The amplitude of rapid drop, similarly to the case of 700 kPa, increases and decreases alternately. In the case of a sample built of new beads, the maximum value of that amplitude is higher than in the test with beads used once: 478 kPa and 398 kPa, respectively. Repetitive tests on grains used in former experiments revealed lower strength of the material when compared to those performed on virgin beads, which suggests that the tested material becomes weaker with an increasing number of test repetitions. One explanation for this effect is that the glass that makes up the beads can be cracked or crushed on a micro-scale, limiting its strength. Such grain fatigue is shown in Fig. 5. The upper part of Fig. 5 shows glass grains magnified 100$\times$. In the upper left corner, virgin glass beads are presented, whereas in the upper right corner, reused beads are placed. Small dots are visible on the surface of reused beads. Further magnification (1000$\times$) reveals that the dots may be fragments of crushed glass, resulting from grain damage during previous shearing (compare bottom right and left parts of Fig. 5). Grain crushing alters the particle structure, leading to changes in surface roughness and the presence of fine crushed fragments within the sample. As a result, the material’s friction angle decreases, reducing the overall sample strength.

Similar results were obtained by Cui et al.^22^. Tests carried out for three values of confining pressure: 200, 400 and 600 kPa show that reused beads used in the tests are not prone to develop the stick–slip events, and such samples have lower strength than those built of virgin beads. Their observations under the microscope revealed that individual beads contain surface scratches after shearing. The stick–slip events also influence the course of the volumetric strain curve, as shown in Figs. 2 and 4. However, the extent of this influence is significantly lower than in the case of stress-strain plots.

The changes in volumetric strain, when considering the envelope curve and ignoring sudden stress drops, are characteristic of those reported for compacted sand samples. Namely, the initial phase of compaction is followed by significant dilation. Furthermore, Figs. 2 and 4 manifest the influence of the degree of wear of glass beads on the volumetric strain level. The virgin beads exhibit initially lower compaction than used beads, followed by notable dilation, which in case of confining pressure of 700 kPa is comparable to those of used glass beads (Fig. 2), but is more pronounced in case of confining pressure of 800 kPa (Fig. 4). The stick–slip events observed in the volumetric strain plot correspond to the points where deviatoric stress drops occur.

**Fig. 6:**
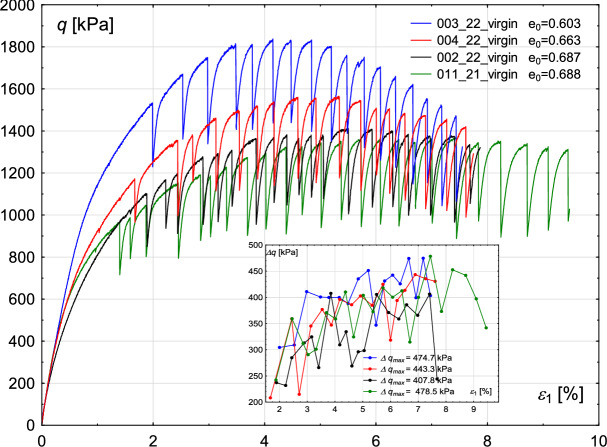
Sensitivity of the experimental results to the value of the initial void ratio. Deviatoric stress *q* (main figure) and deviatoric stress amplitude $\Delta q$ (inner figure) with increasing axial strain $\epsilon _{1}$.

It follows that the rapid drop of deviatoric stress causes the relaxation of the material, so the released force leads to an increase in deformation (Fig. 6).

In the case of the confining pressure of 700 kPa, the oscillation amplitude, i.e “distance” between stick–slip events in $\epsilon _{1}$ alternately increases and decreases until the maximum strength of the material is reached, see inner figure in Fig. 2. After reaching the maximum value of the deviator, oscillation amplitude $\Delta \epsilon _{1}$ decreases drastically, which means that the phenomenon occurs more frequently, but at the cost of a decrease in the deviator amplitude. In the case of the confining pressure of 800 kPa, the oscillation amplitude $\Delta \epsilon _{1}$ increases and decreases sequentially, see an inner figure in Figure 4. For virgin beads, its maximum value is smaller than for beads used once (0.58% and 0.66%, respectively).

Series of subsequent sensitivity analyses, presented in the following subsections, were performed on samples built of virgin beads and aim to determine the extent to which various test conditions influence the results.

### Initial void ratio

**Fig. 7:**
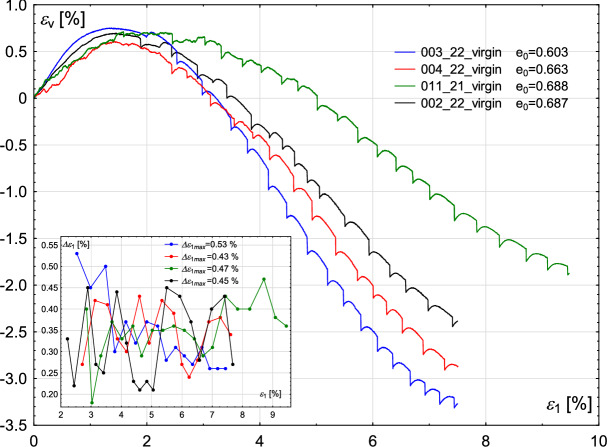
Sensitivity of the experimental results to the value of the initial void ratio. Volumetric strain $\epsilon _{v}$ (main figure) and oscillation amplitude $\Delta \epsilon _{1}$ (inner figure) with increasing axial strain $\epsilon _{1}$.

Figure 6 presents the sensitivity of the results to the changes in the initial void ratio of the sample. All tests have been performed on virgin beads, with a confining pressure of 800 kPa and a comparable shear rate (*v* is in the range of 15.0–16.4 mm/h). It can be seen that the relatively small decrease of the void ratio (e.g. from 0.688 to 0.603) leads to a significant increase in the sample strength, i.e. from about 1550 kPa to over 1800 kPa. This observation is consistent with classical soil behaviour when compacted sands have higher strength than loose sands and exhibit peak strength. Furthermore, for more compacted samples, the more pronounced peak in Fig. 6 can be observed. For those samples, excluding experiment 002/22, the first stick–slip appears for a higher $\epsilon _{1}$ value. Similar conclusions can be found in^30^. They sheared glass beads in plane strain conditions at low pressures of 50 kPa and 100 kPa with a shear rate of 0.1 mm/min. The corresponding evolution of the volumetric strain is shown in Fig. 7. The level of initial compaction is similar for all the considered cases. Further dilation, however, is higher for samples with lower void ratios.

The amplitude of the rapid drop of deviatoric stress is similar to that in the previous cases. The increase and decrease can be observed alternately. There is no visible relationship between the maximum stick–slip amplitude and the initial void ratio, see the inner figure in Fig. 6. The maximum amplitude for the sample with the lowest void ratio is $\Delta q_{max}=475$ kPa, whereas for the highest void ratio: $\Delta q_{max}=479$ kPa, which makes the values comparable.

Similarly, no unique relationship can be determined between sample compaction and the oscillation amplitude $\Delta \epsilon _{1}$, see inner Fig. 7. The maximum oscillation amplitudes $\Delta \epsilon _{1}$ in all four experiments are in the range 0.45–0.53%, where the highest value corresponds to the sample with the lowest void ratio ($e_{0}=0.603$).

### Shear rate

**Fig. 8:**
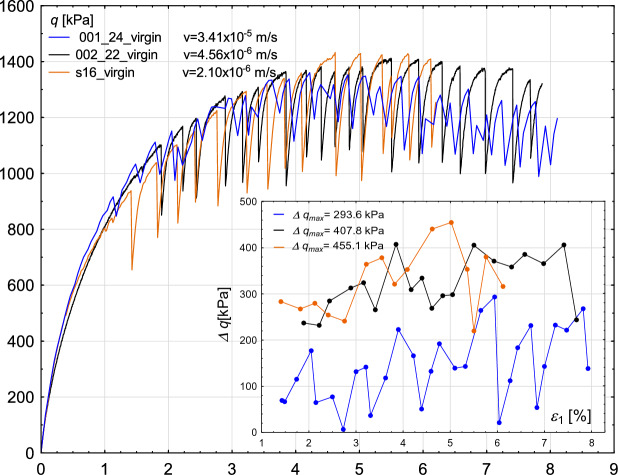
Sensitivity of the experimental results to the value of the shear rate. Deviatoric stress *q* (main figure) and deviatoric stress amplitude $\Delta q$ (inner figure) with increasing axial strain $\epsilon _{1}$.

Figure 8 presents the stress–strain response from experiments conducted on samples composed of new beads, subjected to different shear rates. In all tests, the confining pressure was maintained at 800 kPa, and the void ratio was kept at a similar level. The results indicate that maximum sample strength exhibits minimal dependence on shear rate. Likewise, shear rate does not influence the onset of stick–slip events. While some variation is observed in the timing of the first stick–slip occurrence, no clear relationship emerges. Notably, the amplitude of the deviatoric stress drop decreases with increasing shear rate (e.g., $\Delta q_{\text {max}} = 455$ kPa for the lowest velocity, and $\Delta q_{\text {max}} = 294$ kPa for the highest).

When the sample is sheared with a low rate, the release of energy during a stick–slip event, resulting in both horizontal and vertical deformations, delays the formation of new force chains capable of transferring the load. Conversely, when the sample is sheared quickly—i.e., when the actuator moves toward the sample at a sufficiently high speed—the reorganised structure rapidly forms new force chains, preventing a significant stress drop.

**Fig. 9:**
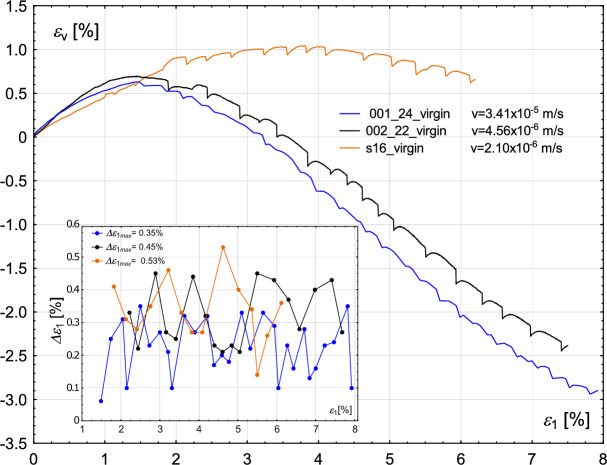
Sensitivity of the experimental results to the value of the shear rate. Volumetric strain $\epsilon _{v}$ (main figure) and oscillation amplitude $\Delta \epsilon _{1}$ (inner figure) with increasing axial strain $\epsilon _{1}$.

Figure 9 shows the evolution of volumetric strain with increasing axial strain. Apart from the test labelled s16 (see Table 2), the influence of shear velocity on volumetric behaviour appears minimal. Tests conducted at lower shear velocities exhibit only minor volumetric changes. After initial compaction, a slight dilation is observed, consistent with the behaviour seen in Fig. 12. In contrast, at higher shear velocities, the dilation phase becomes more pronounced.

Furthermore, the oscillation amplitude $\Delta \epsilon _{1}$ (as shown in the inset of Fig. 9), exhibits a clear dependence on shear velocity. Specifically, the maximum oscillation amplitude $\Delta \epsilon _{1}$ increases as the shear velocity decreases.

This observation is consistent with the findings of Cui et al.^22^ and Doanh et al.^23^, who also reported that stick–slip behaviour becomes suppressed when the shear velocity exceeds a certain threshold.

**Fig. 10:**
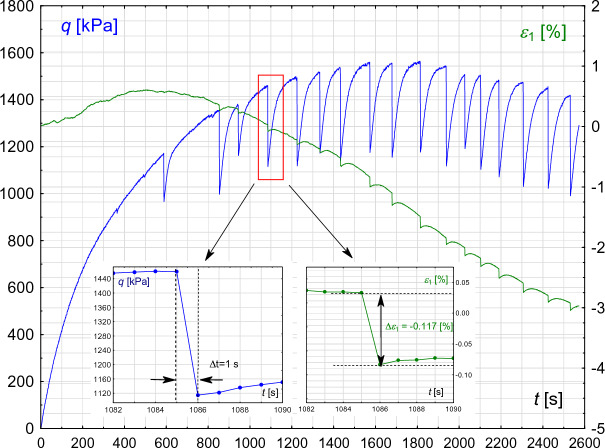
Evolution of deviatoric stress and axial strain as a function of time. The inner figures provide a magnified view of the selected event, highlighting its duration (left) and the corresponding change in axial strain (right).

Figure 10 shows an example experiment 004/22 where the stress deviator changes with time. The inner figures show a magnified stick–slip episode with detailed values: slip time $\Delta t=1$ s (left inner figure) and increase in vertical strain $\Delta \epsilon _{1}=-0.117$% (right inner figure). The time resolution of data acquisition is 1 s, meaning that the only definitive conclusion from this recording frequency is that the slip duration, $\Delta t$, does not exceed 1 s. The slip time issue is thoroughly examined in^22^, where the relationship between event duration and shear rate is presented.

### Confining pressure

**Fig. 11:**
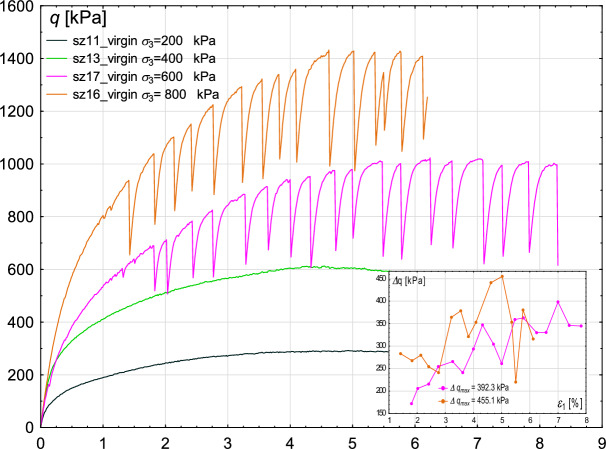
Sensitivity of the experimental results to different values of confining pressure. Deviatoric stress *q* (main figure) and deviatoric stress amplitude $\Delta q$ (inner figure—for cases in which stick–slip events develop) with increasing axial strain $\epsilon _{1}$.

Figure 11 shows the differences in experimental results depending on the level of confining pressure. In all experiments, virgin glass beads were used, while the void ratio and shearing velocity were kept similar. Four confining pressure values were considered: 200, 400, 600, and 800 kPa. The results clearly indicate that the material’s strength depends on the confining pressure and exhibits an increasing trend.

**Fig. 12:**
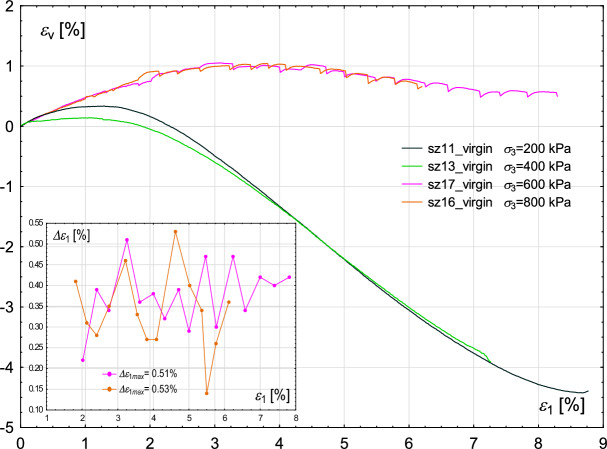
Sensitivity of the experimental results to different values of confining pressure. Volumetric strain $\epsilon _{v}$ (main figure) and oscillation amplitude $\Delta \epsilon _{1}$ (inner figure—for cases in which stick–slip events develop) with increasing axial strain $\epsilon _{1}$.

However, the most interesting observations can be made regarding the stick–slip events. Namely, for the two highest values, i.e. 600 kPa and 800 kPa, stick–slip develops in the entire course of the Fig. 11 plots, starting from relatively low values of axial strain. For the case of $\sigma _{3}$ of 800 kPa, the initiation of stick–slip occurs for a smaller $\epsilon _{1}$ value. Low confining pressure allows the sheared sample to deform freely, but there is a limit value of this pressure above which friction starts to play a role and unrestricted deformation is no longer possible, which manifests itself in the stick–slip effect. The maximum amplitude of the stress deviator drop for confining pressure 800 kPa is higher than in the case of 600 kPa ($\Delta q_{max}=455$ kPa and $\Delta q_{max}=392$ kPa, respectively). The maximum oscillation amplitude $\Delta \epsilon _{1max}$ in both experiments is similar and in the range of 0.51–0.53%, see the inner figure in Fig. 12.

The conclusions from^21,22,29^ agree with observations made based on our results: the stick–slip events are more pronounced at higher confining pressures.

The results presented in this paper, when compared with those of other researchers, indicate that the threshold pressure at which stick–slip events develop depends on both material parameters and test conditions. However, this is a complex problem, and multiple interdependencies have been identified.

First, the shear velocity must be low enough to enable energy accumulation and allow for grain reorganisation. However, the threshold velocity depends on the level of confining pressure: at higher pressures, stick–slip events develop at higher velocities, whereas at lower pressures, the same velocity does not trigger them. This finding is supported when our results are compared with those presented by^19,22,23^.

Additionally, the initial void ratio of the sample plays a crucial role in the development of the stick–slip phenomenon. In loose samples^19,23,24^, the threshold confining pressure required for stick–slip development is lower due to weaker force chains and less structured particle arrangement. However, for more compacted samples with more stable particle arrangements (such as those in our tests), stick–slip emergence requires higher confining pressures. On the other hand, observations recorded in^22^ revealed that stick–slip events also develop in dense samples, for lower confining pressures (e.g. 400 kPa), considering shear velocity comparable to that applied in our tests. Therefore, another factor must exist that shifts the threshold of stick–slip development to lower values in that case. One of the possible reasons is that experiments described in this manuscript were performed in plane strain conditions. As a consequence, the threshold pressure for stick–slip development may differ from that in standard triaxial conditions due to the constrained direction of strain evolution. Furthermore, the force chain network evolution in plane strain conditions is different from that in standard triaxial conditions, which is evidenced in Fig. 20.

Consequently, the corresponding evolution of the volumetric strains with the proceeding axial strain is shown in Fig. 12. For the two cases with the highest confining stress, the evolution of volumetric strain is very different from that obtained for lower values. Namely, the volumetric strain increases slightly, denoting the compaction of the sample, and then starts to decrease, reaching almost the initial level. Overall volumetric changes are minor. The situation is substantially different for the cases with confining pressures of 200 and 400 kPa. After the initial minor increase in volumetric strain, samples start to dilate significantly. Similar observations are made by Cui et al.^22^, where the initial compaction of the sample is more pronounced as the pressure in the chamber increases. Furthermore, in the study by Alshibli and Roussel^40^, the sample exhibited minor initial compaction followed by dilative behaviour. The behaviour of the most confined samples in Fig. 12 differs significantly from that observed in Fig. 7, despite both cases having the same confining pressure of 800 kPa. This difference is most likely due to the variation in shear velocity. Specifically, the shear velocity used in the test shown in Fig. 7 is twice as high as that in Fig. 12. At higher shear rates, the sample tends to exhibit significant dilation, whereas at lower rates, only minor changes in volumetric strain are observed.

### Accumulation and release of energy in stick–slip event

Magnitude of stick–slip event can be considered as an amount of accumulated and released energy. For the selected stick–slip episode, the proportion between the maximum slip energy and the accumulated energy can be assessed according to the formula, see^21^:

1 $$\begin{aligned} k = \frac{S_{2}\Delta q_{2}\Delta d_{2}}{S_{1}\Delta q_{1}\Delta d_{1}}, \end{aligned}$$

where $S_{1}$, $\Delta q_{1}$, $\Delta d_{1}$ are: slip surface area, deviatoric stress increment and vertical displacement, respectively, in the energy accumulation phase. $S_{2}$, $\Delta q_{2}$, $\Delta d_{2}$ characterise the corresponding parameters in the slip and energy release phase.

**Fig. 13:**
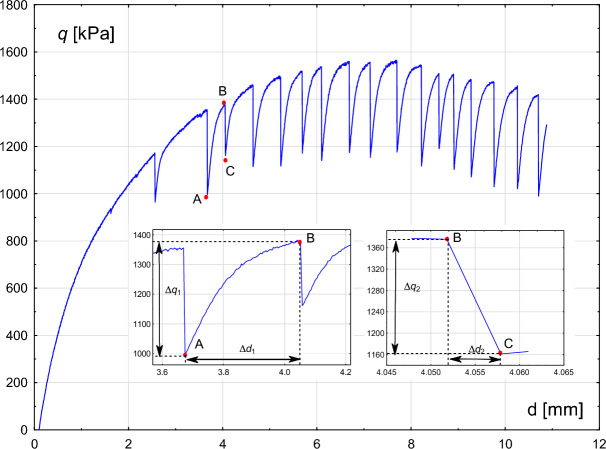
Experiment 004/22: the evolution of the deviatoric stress *q* with increasing axial displacement *d*. Path AB represents the energy accumulation phase (left inner figure), while path BC corresponds to the energy release phase (right inner figure).

For the selected stick–slip event (experiment 004/22), as shown in Fig. 13, points A and B correspond to the accumulation phase, while points B and C represent the relaxation phase. The parameter value ($k = 0.025$) is determined, which is higher than that reported in^21^. However, since *k* remains much smaller than 1, this indicates that the released energy is significantly greater than the accumulated energy. The ratio between $\Delta q_{1}$ and $\Delta q_{2}$ equals 1.75.

Furthermore, the moment of deviatoric stress drop is accompanied by an acoustic emission. This effect was also noted in^21,22^.

## Numerical simulations

The experiment performed in the true triaxial apparatus and described in section 3 is modelled using LIGGGHTS Open-Source Discrete Element Method Particle Simulation Software^41^. Several contact models are implemented in LIGGGHTS to reproduce, to the highest extent, the behaviour of natural grains. In the case of glass beads, the Hertz-Mindlin contact model seems to be the appropriate choice. The deformation of two colliding spheres is reflected in the model by particle overlap. From the magnitude of such an overlap (and thus the distance between particle radii), the value of a contact force is obtained. The contact force consists of normal and tangential components (each includes spring and damping force). The Coulomb friction limit is applied to the magnitude of the tangential component.

The calculations were performed using the high-performance computer Tryton Plus available at TASK Computer Centre in Gdańsk.

### Numerical sample

**Fig. 14:**
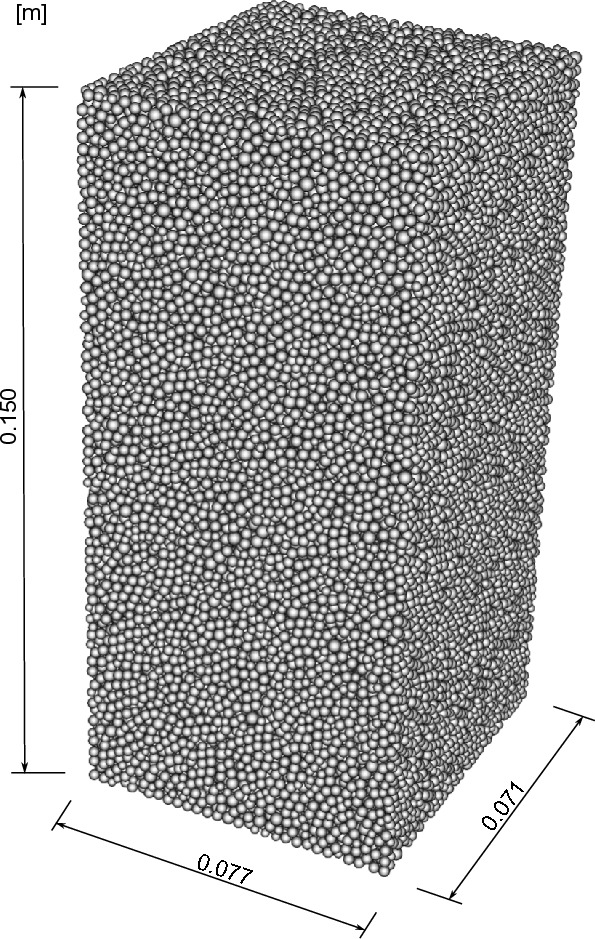
Geometry and grain arrangement of the numerical sample.

The model dimensions correspond to the dimensions of the triaxial specimen described in section 3.1, and are presented in Fig. 14. The set of parameters used in the numerical modelling is shown in Table 3. The characteristic values of Young’s modulus and Poisson’s ratio for aluminium are applied to the box walls. For the glass beads, most parameters are obtained from the analyses presented in the paper by Świtała et al.^42^. However, Young’s modulus is slightly higher, and the friction coefficient between the glass beads is slightly lower than in the referenced paper, to achieve better agreement between the numerical results and the experimental data. The influence of the latter on the stress–strain plot will be discussed later.

**Table 3:** Material parameters used in numerical modelling (reference case).

Parameter	Value	Unit
Young modulus		
Glass beads	5.00	GPa
Box walls	69.00	GPa
Poisson’s ratio		
Glass beads	0.25	–
Box walls	0.32	–
Coefficient of restitution		
Between glass beads	0.80	–
Between glass beads and box walls	0.50	–
Friction coefficient		
Between glass beads	0.35	–
Between glass beads and metal	0.00	–

Since the triaxial sample is relatively large compared to the diameter of a single glass bead, numerical simulations with particles of the exact size would be very time-consuming. Therefore, we scaled the particles to reduce computational effort. The grains simulated in LIGGGHTS have twice the diameter of those used in the triaxial tests.

The test procedure is described as follows. First, the sample geometry is defined, and the walls of the test box are created and saved in STL format. Particles are inserted into the domain using the insert/stream command. Since the timestep in the DEM simulations must be sufficiently small to satisfy the Rayleigh condition (approximately 20% of the Rayleigh wave speed), the shearing velocity is increased by several orders of magnitude to achieve a strain level comparable to that observed in the experiment. Furthermore, the simulations satisfy the condition for a quasi-static regime, as defined in^43^, in which the inertial number does not exceed 1e−3.

Following the procedure of the plane strain triaxial compression test, the numerical sample is initially compressed to reach the target value of 800 kPa. In LIGGGHTS, it is done via mesh/surface/stress/servo command executed on each wall. However, since it is impossible to control pressure, an appropriate target force is calculated and applied to the centre of mass of each wall. The magnitude of that force depends on the walls’ dimensions, and thus, the final compression calculated for the corresponding wall reaches approximately 800 kPa.

After the compression step, the plane strain conditions are applied. Side plates are fixed in their current positions (servo command is released from left, right, top and bottom plates). The pressure of 800 kPa is kept on the front and back walls (servo command is maintained). To achieve the triaxial loading conditions, a constant velocity is applied to the top and the bottom plates.

Since pressure cannot be directly applied using the mesh/surface/stress/servo command, it is necessary to update the target force during compression to maintain the assumed pressure level. Therefore, based on the displacement of subsequent walls in consecutive steps, the area of each wall is calculated. The updated target force is then determined as the product of the prescribed pressure and the corresponding wall area.

### Comparison between experimental and numerical results

**Fig. 15:**
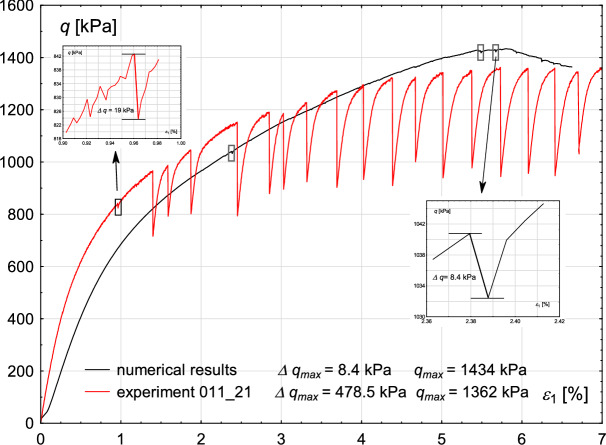
Changes in the deviator stress *q* with proceeding axial strain $\epsilon _{1}$. Comparison between numerical and experimental results. Inner figures present the magnification of the small stick–slip events in the experiment (red) and in the numerical analysis (black), and their amplitude.

The comparison between the experiment and numerical results is shown in Fig. 15. The conditions used in calculations are as follows: particles radii: r=$2 \times r_{exp}$, where $r_{exp}$ represents the radii used in the experiments; shear rate v = 0.52 mm/s, initial void ratio $e_{0}=0.668$ and confining pressure: $\sigma _{3} = 800$ kPa. The numerical results are compared with the experiment no. 011/21, in which the initial void ratio and confining pressure match those used in the numerical analyses, while the shear rate is 0.0043 mm/s.

The maximum strength of the material determined experimentally is lower than the strength resulting from the numerical prediction: $\ q^{max}_{exp}$=1362 kPa and $\ q^{max}_{num}$=1434 kPa, respectively.

The comparison between experimental and numerical results reveals significant limitations of the model in reproducing stick–slip events comparable to those observed in the experiment, particularly in terms of magnitude and frequency. Numerical results show only minor drops in the deviatoric stress, with maximum amplitudes of $\Delta q_{max}=8.4$ kPa (the magnification of such an event is presented in the inner box in the bottom-right corner of Fig. 15). All deviator stress drops with the amplitude greater than 5 kPa are marked with grey rectangles in Fig. 15. The numerical prediction identifies three distinct stick–slip events within the stress range before reaching the limit state. In the experiment, small stick–slip episodes are also observed, with amplitudes comparable to those obtained from numerical results. A selected minor stick–slip event, where $\Delta q_{max}$=19 kPa, is enlarged and displayed in the inner box at the top-left corner of Fig. 15. However, only a qualitative comparison between experimental and numerical results is currently justified.

Theoretically, the Hertz-Mindlin contact model implemented in LIGGGHTS is capable of capturing rapid stress drops caused by stick–slip events. It includes the incorporation of tangential displacement history and applies a Coulomb friction limit, which allows for energy accumulation and release. However, several key factors may explain the difficulties in reproducing stick–slip phenomena to the same extent as observed experimentally.

First, the shear velocity applied in the DEM simulations is higher than that used in the experiments. While a lower velocity would be more physically realistic, it would significantly increase computational time due to the need for small time steps to maintain numerical stability. As a result, higher velocities are typically used, which may prevent proper resolution of the transition from stick to slip. This can also affect the simulation’s ability to accurately represent microstructural changes and grain reorganisation, which are essential for energy accumulation during the stick phase. Consequently, the resulting stress drops may not be comparable to those observed experimentally.

Another limiting factor is the use of a constant friction coefficient for both the stick (static) and slip (dynamic) phases. This simplification may suppress the sharp force drops characteristic of real stick–slip behaviour. A possible solution, to be explored in future simulations, is to differentiate between static and dynamic friction coefficients or to implement more advanced friction models that include, for example, rate-dependent behaviour.

Furthermore, the discrepancies between experimental and numerical results may be attributed to the fact that in the numerical analyses, rigid walls have been applied, resulting in restricted deformations of the sample. In the experimental conditions in which the rubber membrane is used, deformations are less limited.

### Sensitivity analyses

To assess the sensitivity of the numerical solution to changes in shear rate, friction coefficient, and grain size, a series of sensitivity analyses is conducted. In all simulations, the other material parameters, which are not part of the analysis, are set according to the values provided in Table 3.

**Fig. 16:**
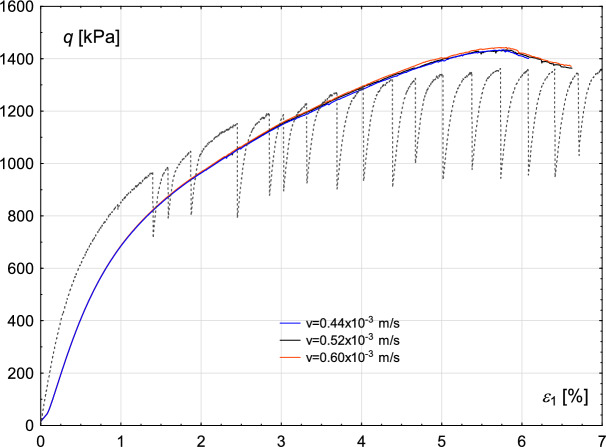
Numerical simulation results illustrating the sensitivity to variations in applied shear velocity. The grey dashed line represents the experimental data.

The sensitivity of the results to changes in shear rate is illustrated in Fig. 16. The dark grey dashed line represents the experimental results. Calculations were performed for three different values of this parameter: 0.44, 0.52 and 0.60 mm/s.

Analysis of the results indicates that material strength is not significantly affected by shear rate, a finding confirmed by experimental studies (see Fig. 8 for comparison). Furthermore, this observation aligns with the widely accepted assumption in soil mechanics that shear rate does not influence material strength.

**Fig. 17:**
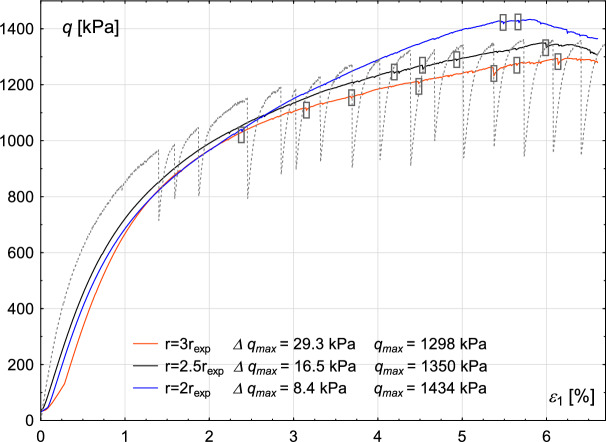
Numerical simulation results illustrating the sensitivity to variations of glass beads radius. The grey dashed line represents the experimental data. Small stick–slip events are marked with black rectangles.

The sensitivity of the results to the glass bead radius, along with a comparison to the experimental results (represented by the dark grey dashed line), is presented in Fig. 17. Calculations were performed for three cases, namely: $r=2 \times r_{exp}$, $r = 2.5 \times r_{exp}$ and $r = 3\times r_{exp}$, where $r_{exp}$ represents the grain radius from the experiment. In each case, the initial void ratio and the shear rate are kept unchanged, and material parameters correspond with those listed in Table 3. The analysis of the results shows that the material’s strength is not highly sensitive to changes in the glass bead radius within the initial range of axial strain, up to 3%. However, after 3%, samples made from grains with larger radii demonstrate lower strength. Furthermore, increasing radius also results in a higher number of small stick–slip events (6, 4 and 3 for cases: 3$r_{exp}$, 2.5$r_{exp}$ and 2$r_{exp}$, respectively). The best fit of the numerical to the experimental results is reached for the radius equal 2.5$r_{exp}$. Experimental studies by Adjemian and Evesque^19^ demonstrate that shear rate and the ratio $d/D_{0}$, of the grain diameter *d* to the initial sample diameter $D_{0}$, influence stick–slip development and the deviator amplitude. This observation can be attributed to the fact that assemblies containing larger grains also have larger pores. As a result, any micro-displacement of a grain within the sample may lead to a greater release of kinetic energy, producing more dynamic effects. Consequently, the rapid drop in strength becomes more pronounced.

**Fig. 18:**
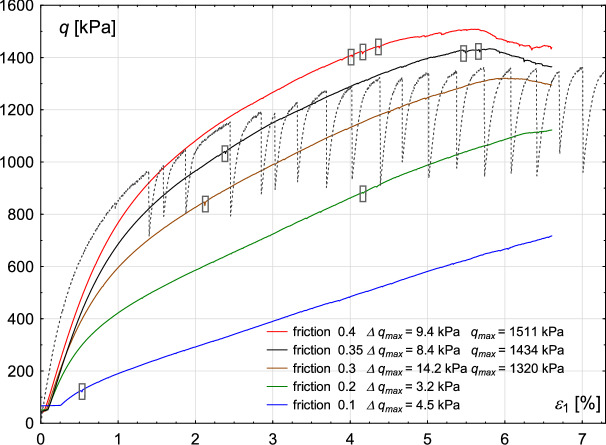
Numerical simulation results illustrating the sensitivity to variations of the friction coefficient of the material. The grey dashed line represents the experimental data. Small stick–slip events are marked with black rectangles.

The sensitivity of the numerical results to variations in the friction coefficient, considering five different values (0.1, 0.2, 0.3, 0.35, and 0.4), is illustrated in Fig. 18. The dark grey dashed line represents the experimental results.

Analysis shows that increasing the friction coefficient leads to an increase in sample strength. A similar effect is observed in the experimental results when the degree of wear of glass beads is considered (see Figs. 1 and 3). This suggests that repeated tests on the same material contribute to its wear, which in turn reduces the friction coefficient. The best agreement with the experimental results is achieved for a friction coefficient of 0.35.

Another observation based on the results presented in Fig. 18 is that a higher friction coefficient leads to the development of more stick–slip events in the stress-strain plot. This trend is also observed in the experimental results, where the degree of wear was considered.

### Micro-mechanical analyses of the numerical sample

**Fig. 19:**
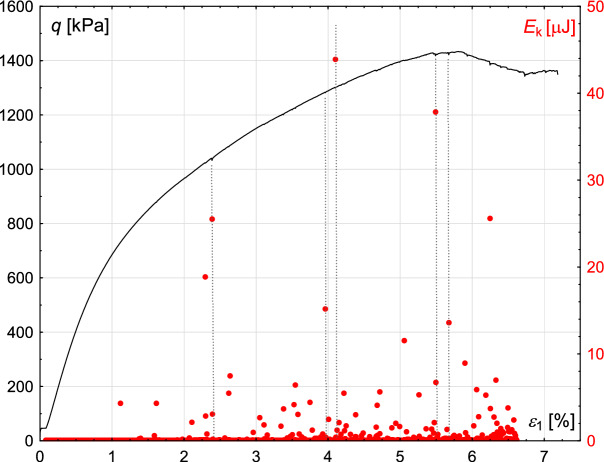
The evolution of the deviator stress with proceeding axial displacements in the numerical analysis (black curve and left vertical axis), and corresponding changes in the kinetic energy (red dots and right vertical axis).

First, the correlation between the macro- and micro-response of the granular assembly is presented. Namely, from Fig. 19, it can be seen that the occurrence of stick–slips is strongly related to the outbursts in the total kinetic energy of the sample. This observation can most likely be attributed to the rapid reorganisation of grains’ structure during stick–slip events, leading to an increase in their velocity and, thus, kinetic energy. Furthermore, from the micro-mechanical point of view, such kinetic energy peaks are the results of the collapse of large voids in the granular assembly^6,42,44,45^. Such collapses may initiate macro-scale events such as stick slips, influencing the course of the stress-strain plot.

**Fig. 20:**
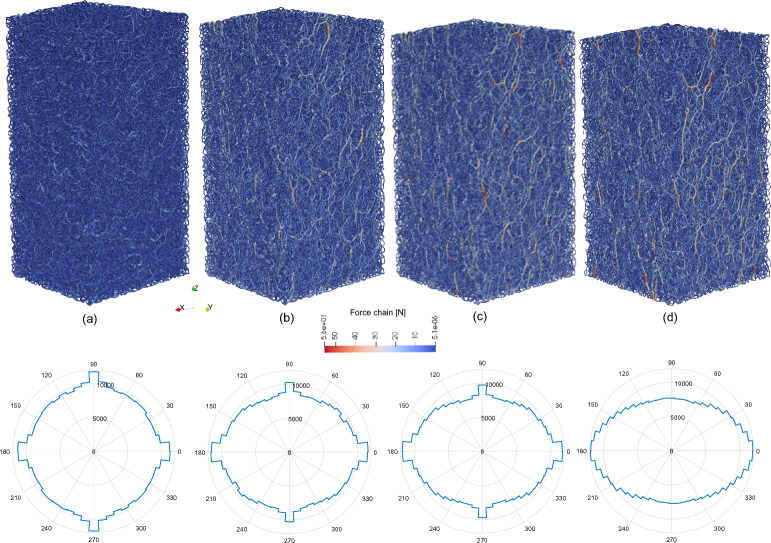
Force chain evolution and contacts distribution during the shearing phase of the triaxial test: (**a**) beginning of shearing, (**b**) 2.45 % axial strain, (**c**) 4.91% axial strain, (**d**) 7.36 % axial strain, end of the test. In polar plots, radial axes correspond to the number of contacts in the model, oriented according to the angles represented by circumferential axes.

The paths of load transmission, called ’force chains’, can be easily visualised in DEM simulations. Therefore, the software Paraview^46^ is used to post-process the DEM results and to investigate the evolution of the complex force chain network in successive shearing stages of the triaxial test (Fig. 20). Figure 20a shows the initial force chains distribution, corresponding to the end of the consolidation phase and beginning of shearing; whereas Fig. 20d presents the final network, at the end of shearing. Throughout the shearing process, new force chains emerge, creating a highly complex structure that continuously evolves and transforms as displacement progresses.

It can be expected that after the uniform consolidation phase, the force chain network is dispersed, and the force distribution is rather isotropic. During the shearing phase, stronger contacts develop in the vertical direction, aligned with the loading direction. However, because in plane strain conditions displacements on the walls perpendicular to the “x” axis are blocked and serve as support for some of the force chains, contact anisotropy in this direction is more pronounced. This phenomenon is confirmed by polar plots showing the distribution of contacts in different directions on the XY plane. Each plot is divided into 50 bins, inside which the number of contacts corresponding to each orientation range is added. These counts are presented on radial axes.

It is possible that, when a stick–slip event occurs, force chains break and are no longer able to carry the load. As the force chains are destroyed, the accumulated energy is released and results in larger deformation. Continued loading of the sample, now with a new structure (as the sample is subjected to a constant deformation rate), leads to the formation of new force chains. These new chains will also eventually break, triggering another stick–slip episode.

Information obtained from DEM analyses, specifically regarding the distribution, orientation, and magnitude of contact forces in the granular assembly, enables a more detailed investigation into the evolution of contact anisotropy, coordination number, and the sample’s state, as reflected in the redundancy factor.

A fabric tensor is a measure commonly used to reflect the anisotropy of contact orientations. A vector defining a contact between two grains is decomposed into individual components corresponding to the directions in the domain, and unit norm vectors ($n_{i}^{c}$,$n_{j}^{c}$) are calculated in each direction. The fabric tensor is defined as^47^:

2 $$\begin{aligned} F_{ij} = \dfrac{2}{N_{p}} \sum _{c \in C} n_{i}^{c} n_{j}^{c} \end{aligned}$$

$N_{p}$ is the number of particles in the considered granular assembly. The sum is over all contacts *c* in the set *C*.

The degree of contact anisotropy can be calculated in 3D from the following formula,^48^:

3 $$\begin{aligned} A = \sqrt{ \left( \left( F_{1} - F_{2}\right) ^{2}+ \left( F_{2} - F_{3}\right) ^{2}+ \left( F_{1} - F_{3}\right) ^{2}\right) /2} \end{aligned}$$

**Fig. 21:**
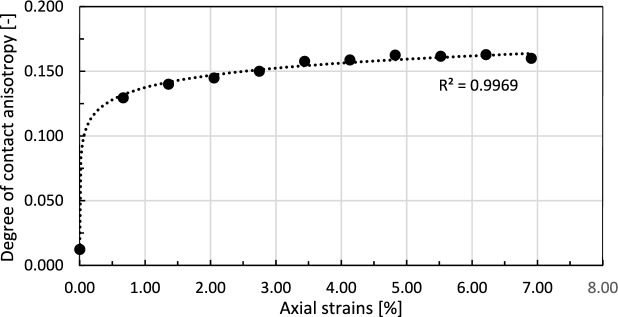
Development of contact anisotropy in the numerical sample with progressing axial strains during the shearing stage of the triaxial compression test. The dashed line represents a fitted logarithmic trend line.

The development of the contact anisotropy is presented in Fig. 21. Initially, after the consolidation phase, the sample is approximately isotropic. This state is also reflected in Fig. 20a, showing the contact distribution. However, as vertical strain increases, the degree of contact anisotropy also rises, eventually reaching a plateau at approximately 0.16. This indicates that the granular structure has stabilized and that transformations within the force chain network become less pronounced.

Another parameter that describes the state of the granular sample is the coordination number, which can be defined as the average number of contacts $N_{c}$ per particle,^47^:

4 $$\begin{aligned} \Gamma = \dfrac{2N_{c}}{N_{p}} \end{aligned}$$

**Fig. 22:**
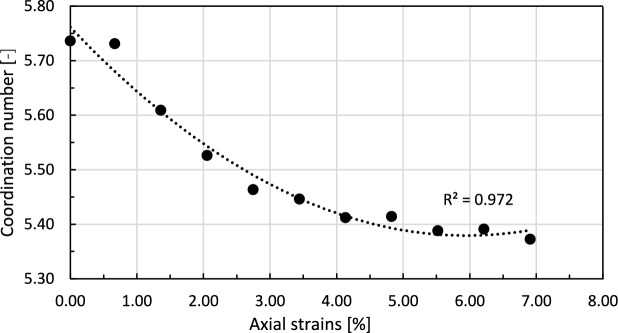
Changes in the coordination number of the granular assembly with progressing axial strains during the shearing stage of the triaxial compression test. The dashed line represents a fitted 2nd-degree polynomial trend line.

The evolution of the coordination number in the course of shearing is presented in Fig. 22. Right after the compression stage of the test, the coordination number of the assembly reaches about 5.75. The contact force network is stable, and particles are densely packed. With progressing axial strain, this parameter gradually decreases, reaching the plateau when the strain level is about 4%. Such a decrease can be attributed to the breakage of some contacts during the shearing and reorganisation of the force chain network. At the end of shearing, the granular assembly recovers the stable state and leads to the particles’ configuration, which can sustain applied stress without significant changes in the number of contacts.

The last measure enables quantifying the excessive constraints concerning the total number of degrees of freedom in the assembly. This parameter can be defined as follows^49^:

5 $$\begin{aligned} RF = \dfrac{N_{c}}{N_{p}} \left( \dfrac{3-2f}{6} \right) \end{aligned}$$

**Fig. 23:**
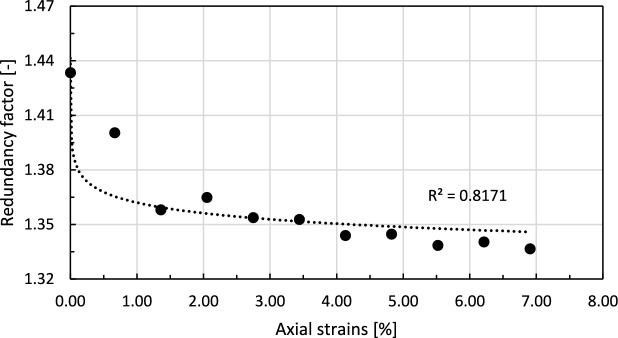
Changes in redundancy factor of the granular assembly with progressing axial strains during the shearing stage of the triaxial compression test. The dashed line represents a fitted exponential trend line.

In the above equation, *f* is the fraction of sliding contacts (in which Coulomb friction is activated) to the total number of contacts. The evolution of the redundancy factor with progressing axial strain is shown in Fig. 23.

The redundancy factor determines whether the sample is in a liquid-like (RF < 1), or a solid-like (RF $\ge$ 1) state. In the considered case, the granular assembly remains a redundant system in a solid-like state throughout the simulation. During shearing, the redundancy factor decreases, following a trend similar to that of the coordination number (see Fig. 22). Near an axial displacement of 4%, this parameter stabilises, reaching an approximately constant value of 1.34.

## Conclusions

A series of experiments on dry glass beads was conducted under plane strain conditions, focusing on observations of the stick–slip phenomenon while considering various material and test parameters. Importantly, analysis of the experimental results reveals that similar factors govern the nature of stick–slip events induced in both plane strain and standard triaxial conditions.

All experimental results form a database that can be used to further investigate the nature and characteristics of stick–slip phenomena, which adds to the existing analyses, e.g. by^20,22,23^. Furthermore, this data helps assess broader dependencies of stick–slip occurrence on various material and test parameters, such as confining pressure level, shear rate, and the ratio between grain diameter and sample size. Such insights can be applied to more complex problems and serve as a foundation for the safe design of structures where stick–slip occurrence is expected.

The DEM model is designed to investigate the micro-scale behaviour of a sample composed of glass beads. The best fit between numerical and experimental results is assessed. However, while slight deviator stress drops, potentially corresponding to stick–slip events, are present in the plot, their small amplitude and low frequency of occurrence do not accurately reflect the experimental results. Nevertheless, potential limitations of the DEM model and sources of these discrepancies have been identified, and future research will focus on addressing these issues.

Despite these challenges, the paper demonstrates that DEM modelling provides valuable opportunities for studying the micro-mechanical behaviour of granular assemblies. First, it allows for the visualisation of the development and evolution of the force chain network during the shearing process. Consequently, this complex structure is shown to undergo continuous transformations in response to variable loading conditions. Furthermore, the relationship between stick–slip events and sudden increases in the specimen’s kinetic energy can be examined. Information obtained from DEM modelling can also be used to determine other micro-scale parameters that provide insight into the sample’s state, which can then be translated into an understanding of its macro-response.

Although many aspects still require further improvement and investigation, we believe that an important first step has been taken in successfully capturing the stick–slip phenomenon in numerical analyses.

## Data Availability

The datasets analysed during the current study are available in the RepOD repository, https://repod.icm.edu.pl/privateurl.xhtml?token=45689816-4818-4ec2-ac10-54ac061c084c.
